# Pneumomediastinum Mimicking Congenital Pulmonary Airway Malformation

**DOI:** 10.3390/children6090104

**Published:** 2019-09-17

**Authors:** Ranjit I Kylat

**Affiliations:** Department of Pediatrics, College of Medicine, University of Arizona, PO BOX 245073, 1501 N Campbell Avenue, Tucson, AZ 85724, USA; rkylat@gmail.com; Tel.: +1-5206266627, Fax: +1-5206265009

**Keywords:** pneumomediastinum, Congenital Pulmonary Airway Malformation, Cystic Adenomatoid Malformation of Lung, congenital, pneumothorax, ultrasonography, interventional

## Abstract

Pneumomediastinum is the collection of free air in the mediastinum. Its incidence is higher in preterm infants and those on positive airway pressure support or on mechanical ventilation. But it has decreased dramatically after the introduction of surfactant and synchronized, non-invasive mechanical ventilation. Underlying cystic lesions could also increase the risk of pneumomediastinum and other air leak syndromes. Most cases resolve spontaneously but rare hemodynamic compromise may require ultrasound-guided intervention.

## 1. Introduction

Pneumomediastinum (PM) is the collection of free air in the mediastinum [1]. Its incidence in neonates ranges from 1% to 2%, but can be as high as 40% in ventilated neonates [2]. Here we describe a case of PM in a term infant which mimicked a cystic lung lesion and persisted for over 2 weeks. 

## 2. Case Summary

**Figure 1:**
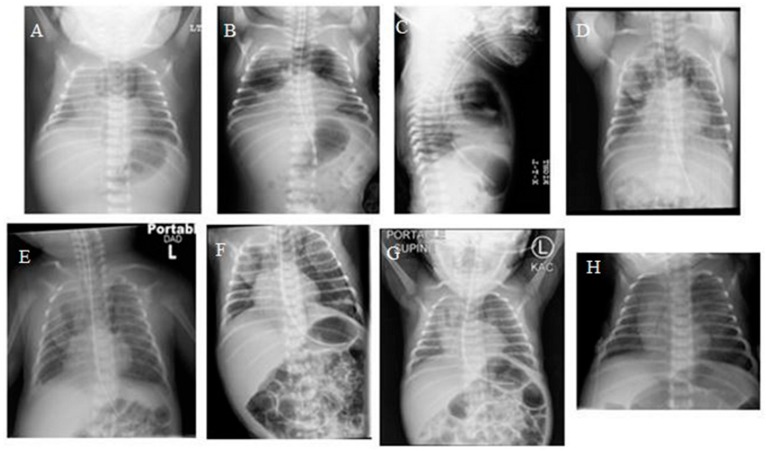
Serial plain chest AP radiograph showing progression of pneumomediastinum (PM) (**A**,**B**,**D**) and resolution (**H**); Lateral chest radiograph showing PM (**C**).

**Figure 2:**
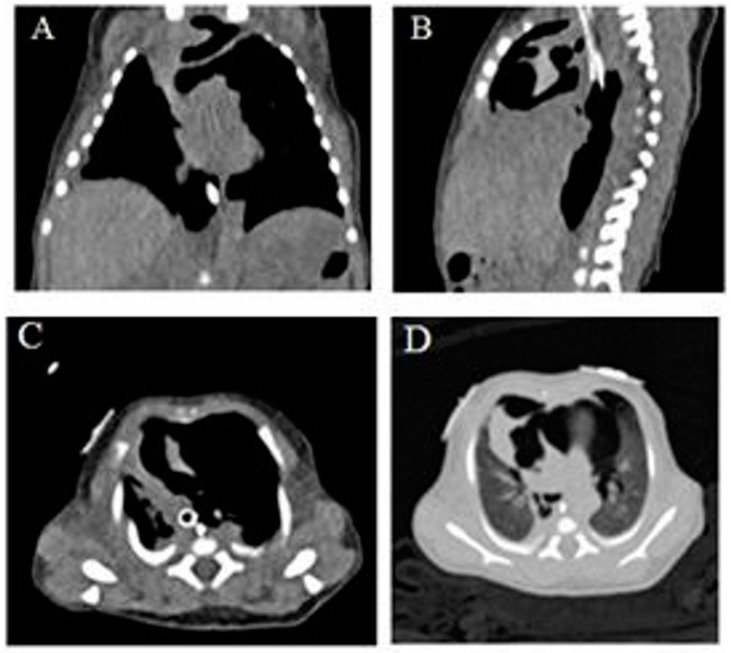
Computed tomograph of chest without contrast showing large PM (**A**–**D**).

A 1-day-old term infant was transferred from an outside facility due to respiratory distress. The infant was born at 37 weeks’ gestation to a 29-year-old woman. Pregnancy was complicated by maternal drug and alcohol use, non-immune hydrops with large right pleural effusion, and mild ascites detected at 22 weeks’ gestation. She had one further visit 1 month later, when hydrops had resolved. She had another child with autism and a prior pregnancy with congenital lung lesion that was terminated. When she presented to the referring hospital at 37 weeks, her labor was induced due to oligohydramnios and suspected fetal distress. The baby did not need resuscitation at delivery but required continuous positive airway pressure due to respiratory distress. The birth weight was 2575 g. Four hours later, due to worsening respiratory distress, the patient was intubated and mechanically ventilated. Chest radiography (CXR) prior to that revealed right pleural effusion, large anterior pneumothorax, and large pneumomediastinum (Figure 1A). The patient was transferred from the referring hospital due to the visualization of a possible lung mass. The patient was ventilated on admission but was extubated after 24 h. The patient continued to need non-invasive continuous positive airway pressure (CPAP) and needed to be re-intubated for incipient respiratory failure a few hours later, but needed only low-peak inspiratory pressures, and was then successfully extubated after a further 36 hours. The patient had serial radiographs due to persistent symptomatology, which documented the evolution with improvement, but not resolution, of the mediastinal air (Figure 1B–G). Echocardiography revealed a large patent ductus arteriosus and enlargement of the right side of the heart. The patient continued to need supplemental oxygen and was slow to establish oral feeding, hence remaining in hospital for 3 weeks. Due to delayed resolution, computed tomography (CT) of the chest was performed, which revealed extensive pneumomediastinum with multiple septations, small left anterior pneumothorax, and small right pleural effusion (Figure 2). Follow-up CXR at 1 month of age showed resolution (Figure 1H). At the last follow-up at over 1 year of age, the patient was asymptomatic with a normal CT image. The author retains parental informed consent for the publication, and the study was approved by the institutional ethics research committee.

## 3. Discussion

PM occurs when air escapes from the lung into extra-alveolar spaces generally due to rupture of an over distended alveolus. The over distension can manifest as pulmonary interstitial emphysema, and if the air dissects along the perivascular connective tissue sheath toward the hilum, this can result in a PM, or a pneumothorax if the air goes into the pleural space. It could also lead to subcutaneous emphysema, pneumopericardium, and rarely, pneumoperitoneum. The incidence of PM is higher in preterm infants, particularly those on positive airway pressure support or on mechanical ventilation, and also in those with underlying pulmonary parenchymal diseases such as cystic lesions or pulmonary hypoplasia, or after meconium aspiration syndrome. Apart from alveolar rupture, the perforation of esophagus, trachea, or main bronchus, and the dissection of air from the neck or abdomen are less frequent in the etio-pathogenesis. In infants and children, a common cause of PM is severe bronchial asthma, and rare causes include airway obstruction due to foreign body, traumatic intubations causing esophageal and tracheal injury, pneumonia, and chest trauma [3]. The incidence, of PM has decreased dramatically especially in preterm infants after the introduction of surfactant, improved management of meconium stained amniotic fluid, and synchronized and non-invasive mechanical ventilation [4,5]. The actual incidence in term infants is unknown, as these infants can be asymptomatic and may not be diagnosed. With the neonatal resuscitation guidelines of 2015 encouraging early use of CPAP in the delivery room for both term and preterm infants, there is now an increase in the PM incidence among preterm infants [6].

The clinical symptoms can range from asymptomatic to tachypnea, cyanosis, and respiratory failure. Findings on examination of distant heart sounds in a tachypneic newborn infant may be present. Radiographic signs include a halo of air around the heart, and retrosternal or superior mediastinal radiolucency. The lateral displacement of the mediastinal pleura, the superior elevation of the thymus, known as the spinnaker sail sign or angel wings, and the continuous diaphragm sign are other characteristic signs [7]. This should not be confused with the thymic sail and wave signs, which are both normal findings [8]. Low described that the multiple septa seen within the pneumomediastinum on CT may simulate a ‘bubbly’ lung lesion as seen in congenital pulmonary airway malformation (CPAM) or congenital lobar emphysema, and postulated that it could represent the anatomically known fascia surrounding the thymus [7]. It could also be that the air in the mediastinum often loculates locally and does not dissect widely. Given that underlying cystic lesions can predispose to air leaks, it may be prudent to evaluate for that; however, as seen in this case, the presence of septations could be confused with CPAM. CPAMs generally need resection and can be difficult to distinguish radiographically from malignant pleuropulmonary blastomas. PM resolves spontaneously in most infants, but many infants need supportive care including respiratory support, and the challenge is to reduce the risk of further progression into other air leak syndromes [9]. In severe cases causing hemodynamic decompensation, ultrasound-guided percutaneous drainage has been reported [10,11]. Our patient required only brief and minimal respiratory support on both occasions when intubated and had no hemodynamic consequences; hence, she did not require any aggressive intervention to relieve the air. In addition, most of the air collection was in the mediastinum, an area which is considered difficult to evacuate from. Even though the patient received CPAP in the delivery room, the air leak could be due to the presence of mild pulmonary hypoplasia, given that there was a prior history of oligohydramnios and fetal pleural effusion. However, as the patient was subsequently asymptomatic and had normal imaging, it is unlikely that there was an underlying pulmonary parenchymal disease.
